# Evolution of complexity in RNA-like replicator systems

**DOI:** 10.1186/1745-6150-3-11

**Published:** 2008-03-27

**Authors:** Nobuto Takeuchi, Paulien Hogeweg

**Affiliations:** 1 Theoretical Biology and Bioinformatics, Utrecht University, Padualaan 8, 3584 CH Utrecht, The Netherlands

## Abstract

**Background:**

The evolution of complexity is among the most important questions in biology. The evolution of complexity is often observed as the increase of genetic information or that of the organizational complexity of a system. It is well recognized that the formation of biological organization – be it of molecules or ecosystems – is ultimately instructed by the genetic information, whereas it is also true that the genetic information is functional only in the context of the organization. Therefore, to obtain a more complete picture of the evolution of complexity, we must study the evolution of both information and organization.

**Results:**

Here we investigate the evolution of complexity in a simulated RNA-like replicator system. The simplicity of the system allows us to explicitly model the genotype-phenotype-interaction mapping of individual replicators, whereby we avoid preconceiving the functionality of genotypes (information) or the ecological organization of replicators in the model. In particular, the model assumes that interactions among replicators – to replicate or to be replicated – depend on their secondary structures and base-pair matching. The results showed that a population of replicators, originally consisting of one genotype, evolves to form a complex ecosystem of up to four species. During this diversification, the species evolve through acquiring unique genotypes with distinct ecological functionality. The analysis of this diversification reveals that parasitic replicators, which have been thought to destabilize the replicator's diversity, actually promote the evolution of diversity through generating a novel "niche" for catalytic replicators. This also makes the current replicator system extremely stable upon the evolution of parasites. The results also show that the stability of the system crucially depends on the spatial pattern formation of replicators. Finally, the evolutionary dynamics is shown to significantly depend on the mutation rate.

**Conclusion:**

The interdependence of information and organization can play an important role for the evolution of complexity. Namely, the emergent ecosystem supplies a context in which a novel phenotype gains functionality. Realizing such a phenotype, novel genotypes can evolve, which, in turn, results in the evolution of more complex ecological organization. Hence, the evolutionary feedback between information and organization, and thereby the evolution of complexity.

**Reviewers:**

This article was reviewed by Eugene V Koonin, Eörs Szathmáry (nominated by Anthony M Poole), and Chris Adami. For the full reviews, please go to the Reviewers' comments section.

## Background

How complexity can increase through evolution has been one of the most important questions in biology. As is well recognized, the formation of biological organization – be it of protein complexes or of ecosystems – is ultimately instructed according to the genetic information, which is stored as the patterns of nucleotide sequences in genomes. Hence, the above question boils down to how genetic information increases through evolution. This is, however, a one-sided view. The patterns in nucleotide sequences are biologically functional only in conjunction with the organization (e.g., consider the function of a regulatory gene). Thus, organization and information are mutually dependent, and this interdependence is, as this study will show, a key to understanding the evolution of biological complexity.

**Figure 1:**
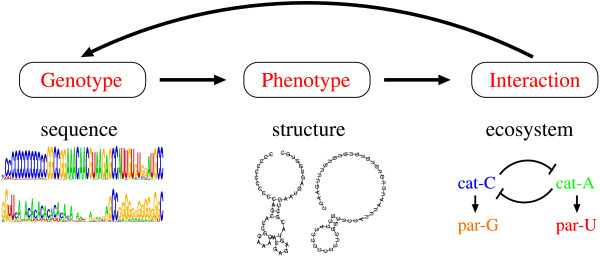
**A schematic representation of the structure of our model**. The arrows represent the direction of causal influence (see main text). The RNA secondary structures depicted under "Phenotype" are constructed from a genotype of the C-catalyst (the left one is from the catalytic strand; the right one is from the template strand) (see main text and Fig. 2 for the C-catalyst). The following can be skipped until Discussion: It is interesting to note that while the direction of causal influence is from information to organization, the direction of functional influence is from organization to information.

We here investigate the role of this interdependence for the evolution of complexity in a simulated RNA-like replicator system. The relative simplicity of RNA-like replicators enables us to explicitly model a biologically relevant genotype-phenotype-interaction mapping of individual replicators, whereby we avoid preconceiving the ecological functionality of genotypes (information) or the ecological organization of replicators in the model. In more detail, the genotype-phenotype mapping is modeled by RNA folding, where the pattern of an RNA sequence (genotype) determines its secondary structure (phenotype) [1]. The phenotype-interaction mapping is constructed such that interactions among RNA molecules – either to replicate or to be replicated – depend on their secondary structures and base-pair matching among their dangling-ends. The interactions among replicators then cause a dynamic feedback on the (spatiotemporal) distribution of genotypes (see Fig. 1). Thereby, evolution in our model occurs as a process of pattern formation in the genotype, the phenotype and the interactions of replicators.

Many theoretical studies have investigated the evolution of complexity in RNA-like replicator systems (e.g., see [2,3] for review, and [4] for the experimental background). The customary approach in these studies has been to assume a network of replicators, where nodes represent replicator species, and edges represent interactions among them. This approach, however, has limitations, in that it leaves unexplained how a multitude of species and their ecological organization emerge from the evolution of individual replicators. For example, a classical study suggested the hypercycle [5], a cycle of cooperatively coupled replicators, as a possible solution to the problem of the error-threshold [6], but it did not demonstrate how the hypercycle can originate. The study was later extended, e.g., by introducing random mutation of interaction strength in various replicator networks [7] or by introducing random growth in replicator networks [8,9]. A new approach has been taken by several studies [10-12], which considered evolutionary processes at the individual replicator level through taking a genotype-phenotype mapping of interacting replicators into account. However, these studies still have the same limitations, since their model assumed a preconceived replicator network (as in the hypercycle study) and the random allocation of phenotypes to the replicator species – hence, organization and information were preconceived.

In contrast, our model explicitly takes account of a genotype-phenotype-interaction mapping of individuals without preconceiving the functionality of genotypes or the ecological organization of a replicator system. Nevertheless, as we will see below, information and organization arise as the system's emergent properties generated by evolving individuals. The main focus of this study is to investigate when and how this happens and, thereby, obtain general insights about the evolution of biological complexity. As a conclusion, we will suggest the following scenario for the evolution of biological complexity: If an emergent ecological organization is established, it can supply a context in which a novel phenotype gains functionality. This promotes the evolution of genotypes realizing such a phenotype – the evolution of novel information. The evolution of such genotypes, in turn, leads to the evolution of more complex ecological organization. Hence, the evolutionary feedback between information and organization.

## Results

### Model

Our model is spatially extended, individual based, Monte Carlo simulation model. It consists of molecules represented by RNA strings of length 50 and their secondary structure determined through RNA folding (Vienna RNA package is used [13]). RNA molecules are located on a two-dimensional square grid of size 512 × 512 with toroidal boundaries, where one square (one spot) holds at most one molecule. The temporal dynamics of the model is run by consecutively applying a reaction and a diffusion step.

In the reaction step, molecules can undergo four types of chemical reactions: (a) complex formation/dissociation; (b) replication; and (c) decay, as designated in Eq. (1).

(1) $$\begin{array}{ll} \text{(a)} & \text{X}+\text{Y}\overset{k_{1}}{\underset{k_{2}}{⇌}}{\text{C}}_{{\text{X}}^{5^{\prime }}\sim^{3^{\prime }}\text{Y}}\text{ and }\overset{{k^{\prime }}_{1}}{\underset{{k^{\prime }}_{2}}{⇌}}{\text{C}}_{{\text{Y}}^{5^{\prime }}\sim^{3^{\prime }}\text{X}},\\ \text{(b)} & {\text{C}}_{{\text{X}}^{5^{\prime }}\sim^{3^{\prime }}\text{Y}}+\emptyset \overset{\kappa }{\to }\text{X}+\text{Y}+{\text{Y}}^{\text{c}},\\ \text{(c)} & \text{X}\overset{d}{\to }\emptyset , \end{array}$$

where X and Y are single molecules; C is a complex molecule; and ∅ is an empty square (interpretable as the resource for replication).

The core part of our model is how to model complex formation and replication reaction, which defines interactions among replicators. Here we employ artificial, yet biologically relevant rules that can incorporate the complexity of RNA folding genotype-phenotype mapping and a high degree of freedom into the phenotype-interaction mapping.

Complex formation happens in our model through binding between the 5'-dangling-end and 3'-dangling-end of two molecules adjacently located on the grid. Binding strength is determined by complementary base-pair matches between the two dangling ends. To be concrete, the probability of binding *k*_1 _is calculated as 1 - *e*^*G *^(that of unbinding is *k*_2 _= 1 - *k*_1_), where *G *(< 0) is the minimal additive score of complementary base-pair matches in the alignment of the two dangling ends without gaps. The score is calculated according to the free energy contributions from base-pairs: a GC contributes to *G *by -0.15; so does an AU by -0.1; a GU by -0.05.

Replication reaction can happen in a complex (C) under a certain condition: the molecule binding with its 5'-dangling-end can replicate the other molecule, if it has a predefined catalytic secondary structure and if the complex is adjacent to an empty square (the resource). The catalytic structure was arbitrarily defined as the one having two hairpin-loops connected by one multi-loop, where the size of loops is allowed to vary (in Shapiro's notation [14], it is written as ((((H)S)((H)S)M)S), where H is hairpin-loop, and S is stem, and M is multi-loop). An example of such a structure is depicted in Fig. 1 (the left structure under "phenotype"). The frequency of random sequences having such a structure is 4.1%. The replication produces a new molecule in the empty square. If no mutation happens, this molecule is complementary to the template. Point mutations are introduced with a probability *μ *per base (no other types of mutations are considered). The probability that replication happens given all the conditions met is set to 1 for simplicity. Finally, all molecules also decay with the probability 0.03.

The diffusion step is implemented as a type of random walk: Each molecule can move randomly to one of the eight adjacent squares if the square is empty.

In summary, molecules diffuse and locally interact. The interactions happen via complex formation and replication, which depend on the dangling-ends and secondary structure of molecules (phenotype), which is determined by their sequence (genotype) (see also Fig. 1). The interactions give a dynamic feedback on the spatiotemporal distribution of genotypes. Thereby, evolution occurs as a process of pattern formation in the genotype, the phenotype and the interactions of replicators. (See Methods for more details of the model.)

Simulations are conducted for various mutation rates by initializing the system with a homogeneous population of a pre-evolved replicator (the purpose of the pre-evolution is to simplify the interpretation of the results; see Methods for details). We then investigate the patterns generated by evolution in various levels. Firstly, we analyze the patterns evolved in populations of sequences through constructing phylogenies. In this we recognize that a population consists of different sequence classes (species) depending on the mutation rate. We then characterize each sequence class through analyzing the patterns in its genotypes and phenotypes. This allows us to infer possible interactions among the sequence classes. Based on these results, we next analyze spatiotemporal patterns in the distribution of replicators, and we reveal complex ecological organization of the replicator system. Finally, we investigate how evolution generates these patterns over time and how mutation rate influences the evolution. We also check the robustness of the results.

### Evolved patterns in populations of sequences

**Figure 2:**
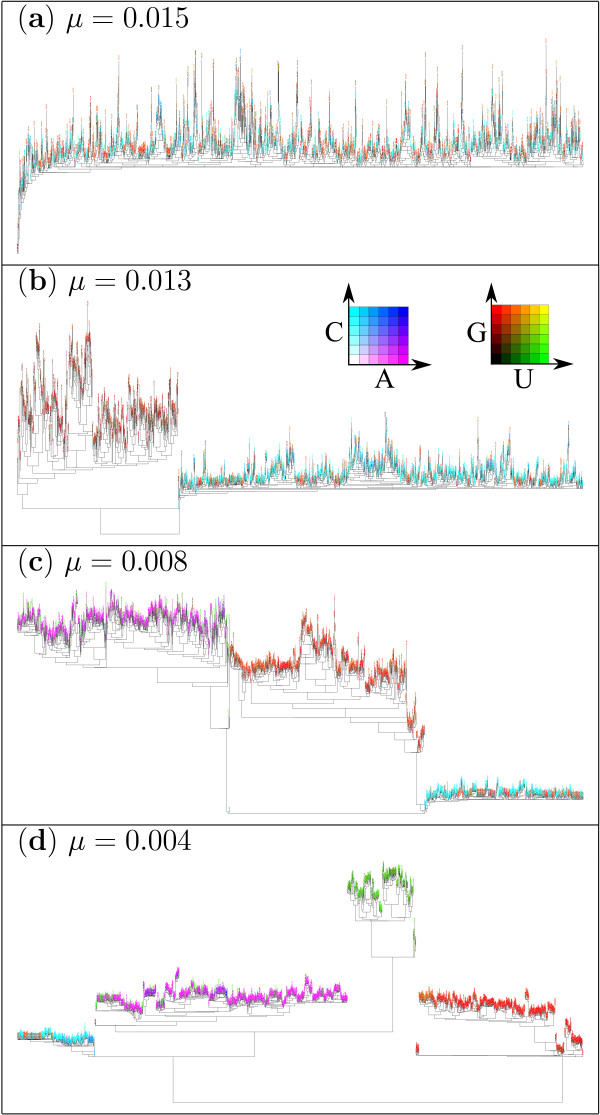
**Phylogeny for various mutation rates**. Phylogenies are constructed from 2000 genotypes selected from a simulation for various values of the mutation rate (*μ*). They were constructed through maximum likelihood method by using PHYML [43]. Due to great divergence among sequences and the procedure in genotype selection, the phylogeny depicts only the patterns in the population of sequences, but not necessarily the evolutionary relationship among them (see Methods for details). The leaves of the phylogenies were colored according to the sequence composition of a genotype's dangling-end. For catalytic genotypes, the 5'-dangling-end of the catalytic strand (which is to recognize templates) is chosen. If the dangling-end has more C's, the color becomes more cyan, whereas, if it contains more A's, then the color becomes more magenta. For non-catalytic genotypes, the 3'-dangling-end (which is to be recognized by catalysts) with the most extreme sequence composition among a pair of complementary sequences is chosen. If the dangling-end has more G's, the color becomes more red, whereas, if more U's, then more green. In this coloring scheme, the C-catalyst tends to appear cyan; the A-catalyst, magenta; the G-parasite, red; the U-parasite, green. However, it should be noted that the red leaves appearing in the clades of the C-catalyst are not the G-parasite (e.g., see (a)). Instead, these represent the mutants of the C-catalyst that have lost the catalytic structure, and they are members of the C-catalyst quasi-species (see also "Evolution of the ecological organization" in main text). This is also the case for the green leaves in the clades of the A-catalyst. Finally, for more precise color coding, insets indicate the colors as a function of nucleotide frequencies, where it reads 0.1 on scales (the frequency more than 0.5 are joined). The left (resp. right) inset is for catalytic (resp. non-catalytic) genotypes.

At a sufficiently later time step in a simulation (ca. 340000th), a phylogeny was constructed to detect patterns in the population of sequences (see Methods for details). Surprisingly, the phylogenies reveal that a complex pattern evolves in a population of sequences depending on the mutation rate (*μ*) as shown in Fig. 2. For a very high mutation rate (*μ *= 0.015), the phylogeny displays no noticeable clade patterns. The population is supported by various sequences, some of which are found to have the catalytic structure, while the others do not. The absence of clade patterns indicate that the population forms one quasi-species [15]. For a slightly smaller value of *μ *(= 0.013), however, the phylogeny reveals the existence of two sequence classes (two quasi-species), which are characterized by their distinct sequence patterns. As *μ *becomes even smaller (*μ *= 0.008, 0.004), the number of sequence classes increases up to four. In addition, for even smaller values of *μ *(< 0.001), the number of evolved sequence classes fluctuates during the simulation between 2 and 5 (the results for this parameter region will not be further dealt with in this paper).

### Patterns in the genotypes and the phenotypes of sequence classes

**Figure 3:**
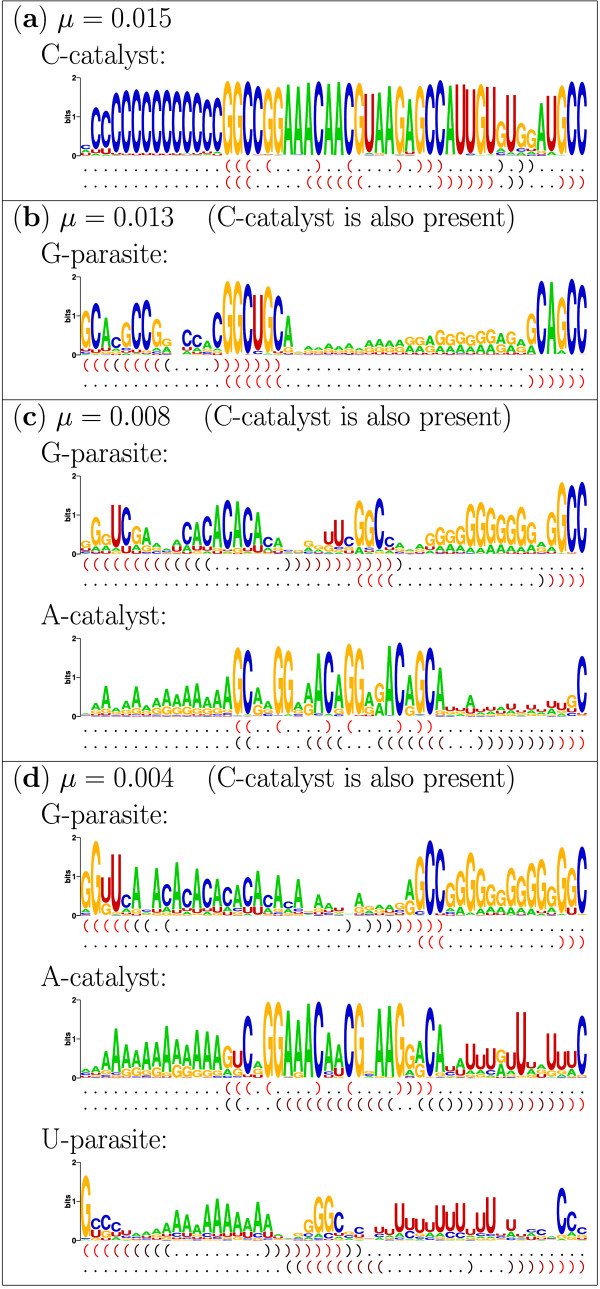
**Sequence logos and typical secondary structures for each sequence classes**. Genotypes were categorized into sequence classes through the phylogenies shown in Fig. 2 (see Methods for mode details). Sequence logos were produced by using WebLogo [45]. The typical secondary structures were produced as follows. For a set of aligned structures represented by dot-bracket notation, the frequencies of bound and unbound bases (i.e., '(', ')' and '.') are counted for each structure position. For each position, if the frequency of '(' or of ')' exceeds 20%, then the corresponding bracket is drawn; otherwise, '.' is drawn. The color of the brackets represents its frequency: the more frequent, the more red. Physical complexity *C *[40] was calculated from the sequence logos (see Discussion for details). For *μ *= 0.004, *C*_*C *_= 41.3 for the C-catalyst; *C*_*G *_= 22.5 for the G-parasite; *C*_*A *_= 26.5 for the A-catalyst; *C*_*U *_= 13.0 for the U-parasite; and *C*_∀ _= 13.4 if all sequence classes are grouped together. For *μ *= 0.008, *C*_*C *_= 41.5; *C*_*G *_= 19.9; *C*_*A *_= 20.9; and *C*_∀ _= 15.7. For *μ *= 0.013, *C*_*C *_= 37.9; *C*_*G *_= 21.0; and *C*_∀ _= 27.5. For *μ *= 0.015, *C*_*C *_= 38.2 (*C*_*C *_= *C*_∀ _by definition).

To characterize these sequence classes, their sequence logo [16] and typical secondary structure are analyzed as shown in Fig. 3.

For *μ *= 0.015, there is only one sequence class. Its sequence and structure have evolved remarkable patterns optimized for its own replication. But to understand this, one has to consider the following. To be a self-replicator, a molecule has to replicate both its exact and complementary copy because replication is complementary (the unit of replication is a pair of complementary sequences). Thus, the 5'-dangling-end of the replicase strand (the strand having the replication activity) must make sufficient base-pair matches both with the 3'-dangling-end of its complementary copy (template strand) and with that of its exact copy. The latter is much more difficult because too many base-pair matches between the dangling-ends in the same sequence can cause intra-molecular binding (the dangling-ends disappear by forming stems), which prevents replication.

How did evolution tackle this problem? As shown in Fig. 3(a), the replicator has a long dangling-end in the 5'-end of the replicase strand and in the 3'-end of the template strand, and these dangling-ends have an extremely high frequency of either C's or G's (note that GC-pairs have the strongest affinity). Moreover, the 3'-dangling-end of the replicase strand also has many G's, but they are interspersed by U's. These U's prevent base-pair formation with its own 5'-dangling-end (intra-molecular binding) by introducing many potential bulges. These bulges are, however, not destabilizing for complex formation due to the artificial interaction rules (see Methods). Finally, the template strand has no 5'-dangling-end, which prevents the formation of non-functional complexes. In this way, evolution optimized – partly by playing on the interaction rules' simplicity – the phenotype of the replicator for self-replication. For convenience, this sequence class is hereafter called C-catalyst.

The phenotype of the C-catalyst described above is remarkable, because it is a very intricate matter to realize it in a viable replicator. As one can see from Fig. 3(a), the base-pairs forming in the secondary structures are almost all GC's. But these G's and C's are "hidden" in the sequence that contains many other G's and C's. This makes the folding of correct structures difficult, and, therefore, the replicator must delicately tune up its sequence. This can be indeed seen from the fact that the genotypes of the C-catalyst are extremely well conserved over all sequence positions despite a high mutation rate (cf. the other sequence classes).

Let us next look at the other sequence classes appearing for lower *μ *values. For *μ *= 0.013, while the C-catalyst also appears in the system, there is another sequence class as shown in Fig. 3(b). Interestingly, this class has no catalytic structure in either strand, but has a long 3'-dangling-end and no 5'-dangling-end in both strands. These secondary structures are achieved through the alternative stacking of three highly conserved sequence regions. The 3'-dangling end of both strands has a high frequency of G's (especially the longer ones), and, moreover, the absence of 5'-dangling-ends prevents the formation of non-functional complexes. These patterns clearly indicate that this sequence class is optimized for being replicated by the C-catalyst (thus named G-parasite).

For *μ *= 0.008, yet another sequence class appears as shown in Fig. 3(c). This sequence class has a structure that is optimized for replication in a similar manner as in the C-catalyst. However, while the C-catalyst uses GC-pairs to form complexes, this sequence class instead uses AU-pairs (thus named A-catalyst). It is likely that this distinctively different sequence composition defends the A-catalyst from the exploitation by the G-parasite (we come back to this point in the next section). Moreover, the sequence of the A-catalyst is much more variable than that of the the C-catalyst, especially at the positions that do not make base-pairs. This indicates that the A-catalyst has a higher degree of freedom in its sequence to maintain the phenotype. This is understood because the bases that form pairs in the secondary structures are mostly G's and C's, while those that do not form pairs are predominantly A's and U's. Finally, it is worth realizing that the system evolves the C-catalyst rather than the A-catalyst for higher mutation rates, even though the A-catalyst has a higher sequence freedom and, thus, is more robust against mutations. This is an interesting counter example to survival of the flattest [15,17] (this will be explained in "Effect of the mutation rate on the evolution").

In addition, note that the sequence pattern of the G-parasite for *μ *= 0.008 drastically differs from that for *μ *= 0.013. These two types of the G-parasite have originated from the same genotype (the population was initially homogeneous), but followed a different evolutionary path (data not shown; the G-parasite sequence for *μ *= 0.013 shown in Fig. 3 is originally the template strand of the C-catalyst, while that for *μ *= 0.008 is originally the catalytic strand of the C-catalyst). They, however, show effectively the same parasitic phenotype albeit achieved by different sequences.

For *μ *= 0.004, yet another sequence class appears as shown in Fig. 3(d). This class is similar to the G-parasite, in that it has long 3'-dangling-ends and no 5'-dangling-ends. Moreover, it achieves its secondary structure through the alternative stacking of three sequence regions as in the G-parasite. But, in contrast to the G-parasite, this class has accumulated a high frequency of U's in its 5'-dangling-ends. This indicates that this class is optimized for being replicated by the A-catalyst (thus, named U-parasite). Therefore, the U-parasite, while achieving the parasitic phenotype in a similar way, has a different "host" from that of the G-parasite.

In summary, the system has evolved complex patterns of sequence and structure, from which multiple sequence classes can be distinguished. Moreover, the observed patterns in the sequence classes clearly indicate catalyst-parasite relationships between them. These relationships imply the evolution of complex ecological organization, which will be further studied in the next section.

### Ecological organization of the replicator system

We saw that four sequence classes can coexist for *μ *= 0.004. In particular, the coexistence between the C-catalyst and the A-catalyst is intriguing since their resource is identical, and, moreover, the sequence patterns they evolved exclude the possibility of mutualistic coupling between them such as the hypercycle. How do they coexist?

**Figure 4:**
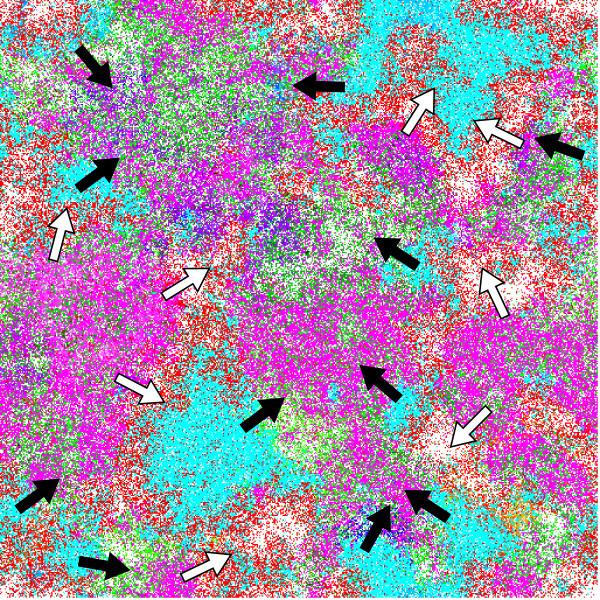
**A snapshot of a simulation**. *μ *= 0.004. The snapshot depicts mutually invading wave patterns. Molecules are colored in the same way as in Fig. 2. In this color scheme, the wave fronts composed of the C-catalyst appears cyan; the wave backs composed of the G-parasite appear red; the wave fronts composed of the A-catalyst appear magenta; the wave backs composed of the U-parasite appear green. Empty space is white. The arrows represent the direction of the propagation of wave fronts. The black arrows are for the C-catalyst fronts, whereas the white arrows are for the A-catalyst fronts. A movie of this simulation is available as Additional file 1.

Fig. 4 shows a snapshot of a simulation, which depicts the spatial distribution of molecules and its temporal dynamics for *μ *= 0.004 (see Fig. 2 for the color coding; see also a movie in Additional file 1). From the snapshot, one can recognize mutually invading wave patterns, where two types of waves exist: one which has the C-catalyst in its front and the G-parasite in its backs (we call it the CG-wave); the other which has the A-catalyst in its front and the U-parasite in its backs (AU-wave). The temporal dynamics of the waves shows that the front of the CG-wave can invade the AU-wave, while the front of the AU-wave can invade the back of the CG-wave. Because of this mutual invasion, both wave types can persist indefinitely, and thereby the four sequence classes can coexist.

The dynamics of spatial patterns clearly indicate the following ecological relationship between the sequence classes: The C-catalyst can out-compete the A-catalyst in the absence of the G-parasite, while the A-catalyst can out-compete the C-catalyst in the presence of the G-parasite. This relationship can be explained by their sequence patterns. On the one hand, the C-catalyst has a rather high frequency of U's in the 3'-dangling-end of the catalytic strand, which makes it possible for the C-catalyst to be replicated by the A-catalyst. But, the A-catalyst has few G's in its 3'-dangling-ends. Therefore, the C-catalyst has an replication advantage over the A-catalyst. On the other hand, the A-catalyst has a different sequence strategy from that of the C-catalyst, which enables itself to escape from the exploitation by the G-parasite. Therefore, if the G-parasite is present, the A-catalyst can invade the C-catalyst. The U-parasite is not necessary for the coexistence between the C-, the A-catalyst and the G-parasite, but it obviously helps the C-catalyst to invade the A-catalyst (this makes the generated spatial patterns much clearer).

We also examined the importance of spatial pattern formation either by repeating or continuing the simulations in a well-mixed situation. It turned out that the system quickly went to extinction in all simulations conducted (data not shown).

In summary, the results of this section revealed the complex ecological organization of the replicator system, and how this organization is generated by the interactions among different sequence classes. Moreover, it was shown that spatial pattern formation is crucial for the evolution of the complex organization and even for the viability of the system.

### Evolution of the ecological organization

So far, we have studied the patterns of sequences and the organization of ecosystems as the outcome of evolution. Next, we focus on evolution itself and study how these patterns and organization are generated through the course of evolution.

**Figure 5:**
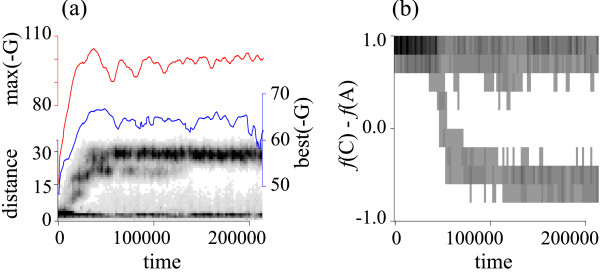
**Dynamics of speciation**. **(a) **Evolutionary dynamics of the G-parasite. The abscissa is time in simulation steps. There are three kinds of ordinate: (1) The ordinate labeled as distance (black) is the Hamming distance between the C-catalyst and non-catalytic molecules that have a stronger affinity to the C-catalyst than to the A-catalyst (for each of the chosen non-catalytic genotypes, the catalytic genotype that gives the strongest affinity to it is used to calculate the distance). The affinity is measured in the *G *score. The distinction between the C- and A-catalyst was done from the sequence composition of their 5-'dangling-end. Gray scale represents the frequency (occurrence) of phenotypes (the more frequent, the darker). The frequency was averaged over ca. 2000 time steps (100 bins). The plots are based on the top 1000 most abundant phenotypes chosen each from catalytic and non-catalytic replicators at every 500 time steps (this is also the case in (b); for the definition of phenotypes, see "Construction of phylogenies"). (2) The ordinate labeled as max(-*G*) (red) is the average of the theoretical maximal affinity of G-parasites (the sequences in the upper branch of the Hamming distance distribution are the G-parasites). (3) The ordinate labeled as best(-G) (blue) is the average of the maximal affinity G-parasites can gain from the C-catalyst. **(b) **Evolutionary dynamics of the A-catalyst. The abscissa is time. The ordinate is the frequency of C's minus that of A's in the 5'-dangling-end of catalytic molecules. Gray scale represents the number of occurrence of phenotypes. The frequency was averaged in the same way as in the Hamming distance in (a).

A simulation for *μ *= 0.01 was analyzed in time series (for *μ *= 0.01, the system evolves the C- and the A-catalyst, and the G-parasite). Fig. 5(a) shows the evolution of the Hamming distance between the C-catalyst and the non-catalytic molecules that have a higher affinity to the C-catalyst than to the A-catalyst. This graph reveals a branching event happening shortly after the beginning of the simulation. The branch with a smaller Hamming distance represents the mutants of the C-catalyst that have lost the catalytic structure. Such mutants also appear in the phylogeny shown in Fig. 2 (e.g., the red leaves appearing in (a)). It must be noted that although some of these mutant genotypes can still be replicated by the catalytic genotypes, they do not constitute a separate parasitic lineage – they are members of the C-catalyst's quasi-species. Moreover, they have on average less affinity to the C-catalyst than the G-parasite as seen from the result presented next. The branch with a greater Hamming distance turns out to be the G-parasite as revealed by the sequence analysis. By following this branch, the affinity (*G*) between the G-parasite and the C-catalyst was measured in time as shown in Fig. 5(a). These plots illustrate the evolution of the G-parasite for an improved affinity toward the C-catalyst. These results show that the origin of the G-parasite was in the C-catalyst, and after the branching the G-parasite rapidly diverged from the C-catalyst, evolving its parasitic strategy. Moreover, the results indicate that the G-parasite has continuity in its lineage. This result supports the notion that the G-parasite forms a distinct quasi-species from that of the C-catalyst, which have been indicated by their distinct sequence pattern revealed by the phylogeny. Based on these results and on the fact that the G-parasite has distinct ecological functionality from that of the C-catalyst as shown above, we call the G-parasite a different "species" from the C-catalyst.

We next studied the evolution of the catalysts by measuring the sequence composition of their 5'-dangling-end (precisely speaking, the frequency of C's minus that of A's). As shown in Fig. 5(b), the result uncovers another "speciation" happening in the catalyst population, which gives rise to the A-catalyst. It is important to note that the speciation of the A-catalyst is subsequent to the establishment of the G-parasite (see also a movie in Additional file 1).

In relation to the notion of species, we also examined the effect of recombination on speciation by introducing a recombination step in the model. The results were qualitatively the same (but see also Authors' response to Reviewer's report 2).

Let us now consider why the G-parasite and the A-catalyst evolve. A previous study [18] has shown that in a replicator system with complex formation (as in the current system), parasites have a replication advantage over catalysts. This is because replicating a template costs to a catalyst some finite amount of time during which the catalyst can not be replicated, and the parasites, which do not replicate other molecules, can spend more time as templates than the catalysts. Thus, the presence of catalysts entails a "niche" (ecological functionality) for parasites. In the current system, the C-catalyst creates such a niche, and this enables the evolution of the G-parasite. Moreover, once the C-catalyst-G-parasite organization is established, it creates yet another niche, i.e., a niche for a phenotype that can escape from the G-parasite. Acquiring such a phenotype, the A-catalyst evolves. Finally, the establishment of such an alternative catalyst, in turn, creates a niche for a phenotype that can parasitize this alternative catalyst. This can cause the evolution of the U-parasite, though it does not happen for *μ *= 0.01 (this will be explained in the next section). Finally, under the current artificial interaction rules, this successive speciation ceases to operate after the system exploits all nucleotide types.

In summary, the results of this section demonstrated a chain reaction of niche generation and speciation in an emergent ecosystem: An emergent ecosystem generates novel ecological functionality for a certain phenotype; this promotes the evolution of a novel sequence class (species) realizing such a phenotype through speciation; and this, in turn, results in the development of a more complex ecosystem.

### Effect of the mutation rate on the evolution

Let us now consider why the number of species depend on the mutation rate *μ*. A previous study [18] has shown that in a spatial replicator system with complex formation, a deleterious mutation that destroys both catalytic and template activity of replicators imposes a great disadvantage to parasitic replicators because of the effective dilution of the system by inert molecules. In the current model, increasing the mutation rate (*μ*) certainly increases the rate of deleterious mutation. Moreover, as we saw above, it is not an instantaneous process that the G-parasite gains its functionality; it is a gradual (albeit rapid) evolutionary process. This means that the G-parasite suffers the disadvantage of deleterious mutation especially at its early stage of evolution, in which the G-parasite has not yet evolved a great advantage over the C-catalyst. Thus, too high values of *μ *prohibit the evolution of the G-parasite. This prohibition to the early phase of evolution is well illustrated by the bistability of the G-parasite evolution: we inoculated the G-parasite, which has already evolved in a system with a lower value of *μ*, to a system with *μ *of which value is slightly too high for the G-parasite to evolve, and observed that the G-parasite can survive (data not shown).

The same explanation can be applied for the mutation rate dependency of the U-parasite evolution (however, the bistability was not observed). Moreover, higher mutation rates prohibit the evolution of the U-parasite also through increasing the sequence diversity of the A-catalyst, which makes it difficult to parasitize the A-catalyst (Fig. 3; see also [7,19]).

**Figure 6:**
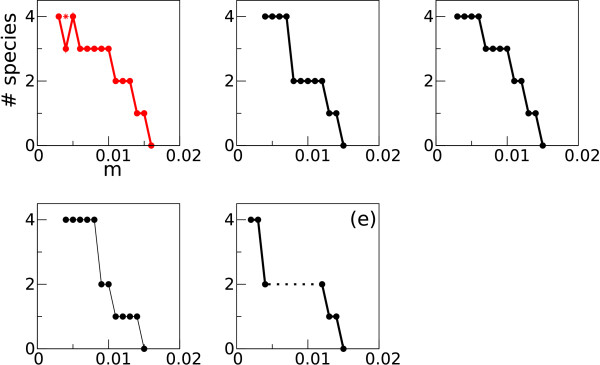
**The number of evolved sequence classes (species) as a function of the mutation rate for five simulation series**. For each series, simulations were initialized with a homogeneous population of different pre-evolved replicators. At some high mutation rate, the number of species drops to zero. This indicates that the system collapses because the mutation rate is above the error-threshold. Plots are not drawn for low mutation rates, because the number of species and their sequence patterns fluctuate in this region of the mutation rate. The red-colored plot is for the simulation series that are described in main text. For *μ *= 0.004, the system eventually evolved to the three species system (the C- and the A-catalyst, and the G-parasite) as explained in main text. The star depicted at *μ *= 0.004 designates the number of species at the metastable state (note that the phylogeny for *μ *= 0.004 in Fig. 2 is generated in this metastable state). The plot designated by (e) is for the simulation series that exhibited functional polymorphism without speciation (the details are explained in Additional file 2). In this plot, the dotted line denotes that the number of species can fluctuate between 2 and 3 (see also Additional file 2).

Finally, the same line of argument can also explain the mutation rate dependency of the A-catalyst evolution. As explained before, the A-catalyst requires the G-parasite for its survival in competition with the C-catalyst (an exception is observed in the system described in Additional file 2). This explains why the A-catalyst – which is mutationally more robust than the C-catalyst – does not evolve for the high values of *μ *for which the G-parasite does not evolve. Moreover, even if the G-parasite evolves, the A-catalyst does not necessarily evolve (see Fig. 3, *μ *= 0.013; see also Fig. 6). This is because greater mutation rates make the G-parasite not very harmful to the C-catalyst, so that the A-catalyst cannot out-compete the C-catalyst in the presence of the G-parasite. But if the mutation rate is sufficiently small, the G-parasite becomes strong enough to enable the A-catalyst to out-compete the C-catalyst in its presence. In this case, the A-catalyst can evolve.

In summary, the diversity of the current system depends on the mutation rate, because greater mutation rates give a disadvantage to replicators with parasitic strategy. Moreover, the above consideration reveals an evolutionary safeguard against harmful parasites: If parasites are sufficiently harmful (because mutation rates are small), escape catalysts evolve exploiting the very harmfulness of the parasite, which stabilizes the system as a whole.

### Stability of the system/Recurrence of the results

The current replicator system is extremely persistent: no single run of simulations showed the extinction of a whole system for *μ *> 0.0001 as long as the initial population survives the first few thousand time steps. Moreover, the ecological organization described above is also stable during a simulation (simulations were continued typically about 500000 time steps [20]). In one case, which is a continuation of the simulation for *μ *= 0.004 described above, we observed that the G-parasite evolved some affinity to the A-catalyst, which made the U-parasite go extinct. However, the G-parasite still had a greater affinity to the C-catalyst, and thus the three species system persisted.

The simulations were repeated for the other pre-evolved replicators. The results turned out to be surprisingly recurrent. From all pre-evolved replicators, the C-catalyst, G-parasite, A-catalyst and U-parasite evolved for small mutation rates (their exact genotypes greatly differed). The mutually invading wave patterns were observed too. Furthermore, as shown in Fig. 6, the relationship between the mutation rate and the presence/absence patterns of the species was qualitatively the same in all cases except for one (see Additional file 2 for this case). Finally, we also examined the effect of spatially heterogeneous decay rates on the evolutionary dynamics and on the formation of spatial pattern by following a previous study [21]. The results were qualitatively the same as presented above (see also Authors' response to Reviewer's report 2).

## Discussion

From a prebiotic evolution point of view, the novelty of the current study is that it demonstrated the evolution of emergent ecological organization in an RNA-like replicator system through the evolution of individual replicators. This contrasts with previous studies [5,7,10,12,21-24], which investigated the maintenance of predefined ecological organization

Moreover, this study sheds new light on the relationship between parasites and the complexity of replicator systems. It has long been thought that the parasites destabilize replicator systems; accordingly, many studies investigated how catalysts can resist or coexist with parasites ([11,18,25,26]; also the references cited in the previous paragraph). Our results, however, showed that parasites can, not only coexist with catalysts, but can actually cause the evolution of further complexity. In fact, Hanczyc and Dorit [27] also observed from their *in vitro *evolution of ribozymes that the system increased its complexity through the evolution of parasites (or partner molecules as they called it), and they suggested that parasites (1) set the stage for the generation of further complexity and (2) be the raw material for the evolution of novel functions. Our results demonstrated the first suggestion, but only partly the second one. The extension of our model to include more kinds of catalytic activities, such as ligase and nuclease, may elucidate this point further (see Ref. [7,28] for evolutionary significance of ligase). In particular, it is of interest whether (or how far) spatial pattern formation alone can support the further increase of functional complexity in the replicator system. It is also of special interest what extra effect – be it positive or negative – can be brought about by the compartmentalization of replicators by membranes [7,22,29,30] or by inorganic materials [31].

Our study also illustrated an interesting relationship between the problem of parasites and that of the error-threshold. For mutation rates close to the error-threshold, the catalysts were free from the evolution of parasitic species, but greater diversity did not evolve either [18]. Lowering the mutation rate caused the evolution of parasites, which, however, enabled the evolution of even greater diversity. Hence, in the current system, greater mutation rates cause smaller replicator diversity, while smaller mutation rates cause greater replicator diversity. Therefore, while it is well known that greater mutation rates limit the accumulation of information due to the error-threshold [6], greater mutation rates can also limit the evolution of complexity by inhibiting the evolution of complex ecological organization.

Finally, we emphasize that extreme persistence was seen in the current spatial replicator system where the evolution at the individual level determines the system's ecological organization, and vice versa. This is because upon the exploitation by harmful parasites, the system can evolve more complex ecological organization by exploiting the very harmfulness of the parasite, which then stabilizes the system as a whole.

From an evolutionary biology point of view, the distinct feature of the current model is that the evolution is modeled as a process of pattern formation in the genotype, the phenotype and the interactions of replicators, where a genotype determines a phenotype; a phenotype determines interactions; and the interactions give a dynamic feedback on the spatiotemporal distribution of genotypes (Fig. 1; a similar view has been taken previously [32]). In the following, we discuss this point through comparison with other models.

Firstly, the current model contrasts with those evolution models where species and their ecological organization are preconceived properties of the system (see also Background). In our model, a species was instead an *a posteriori *recognized entity, representing a multitude of genealogically related individuals (i.e., quasi-species [15]) sharing a distinct functionality in the ecosystem where they lived. Ecological organization was recognized in parallel with that of species through the analysis of the phenotypes and spatial distributions of the species. In other words, species and ecological organization were the system's emergent properties, which evolved as patterns in the genotypes, phenotypes, interactions and spatial distributions of individuals.

Secondly, the current model also contrasts with those models that preconceive the possible ecological functionality that can be achieved by different genotypes (see also Background). For instance, the adaptive dynamics model of [33] predefines a spectrum of resources an organism can consume, and it studies whether/how a population radiates in this space of preconceived ecological functionality (the situation is similar in [34]). In contrast, the ecological functionality of the evolved genotypes in our model is not predefined, but is generated by the ecological organization. Moreover, the ecological organization itself is generated by evolution through a chain reaction of niche generation and speciation. Hence, evolution generates information from within the system.

Finally, the above two points are linked with one another. Emergent organization supplies a context in which novel information can evolve, and the evolution of novel information in turn generates more complex organization. Hence, the positive feedback between the evolution organization and that of information, and thereby the evolution of complexity.

To our knowledge, the only model that has also showed such an auto-catalytic increase in the complexity of an evolving system is Ray's Tierra [35], which studied the evolution of self-copying computer programs. Although it was later reported that the ecological organization in Tierra tended to disappear in the course of evolutionary optimization for self-replication [20], the general insight obtained from Tierra is very similar to that presented above.

Biological complexity has been studied by many authors from various aspects ([36-39] to name a few). Among others, Adami *et al*. [40] suggested to quantify the complexity by the amount of information in sequences (genomes). The information was measured by the degree of conservation in sequences, which is based on the idea that the test for information is how successful sequences are in the environment where they live. Let us consider this in our model. If the complexity (denoted by *C*) is calculated simply from all sequences in the system, it will be lower for the system with more species, contradicting one's intuition. Instead, it seems more natural to calculate *C *for each species separately (as is done in Fig. 3) and then to add them up to the total information in the system. This idea, in fact, shows a view that the functional diversity contributes to the amount of information in a whole system [5]. However, since the functional diversity most likely accompanies sequence diversity, the quantification of information in sequences encounters the dichotomy of conservation and diversity. The question is, how to classify sequences in "right" groups, which depends on how to define what the right groups are. Phylogeny is one of the best tools for this (as demonstrated here), but it is not guaranteed that phylogenetic classes coincide with functional classes (see Additional file 2). This dichotomy may be averted, in principle, by an exhaustive search of all possible sequences for those performing *a priori *specified functions [41]. But there are other problems: First of all, the biological functions of sequences must be discovered with respect to the organization in which they live. Moreover, to sum up the complexity of multiple species (or functions), it may be natural to factor in the functional (dis)similarity between species; however, how to do this quantitatively is an open question.

In closing, the authors note that the general concepts discussed above, such as the evolutionary feedback between information and organization, are not entirely new in the field of evolutionary biology. The point of this study is that our model – where evolution was viewed as a process of multi-level pattern formation – exhibited a prototypical instantiation of those general concepts.

## Conclusion

• Complex ecological organization can evolve in a simple RNA-like replicator system, where parasitic replicators actually promote the evolution of diversity, rather than inhibit it.

• Smaller mutation rates allow the evolution of complex ecological organization, while greater mutation rates inhibit it.

• Extreme persistence is observed in the replicator system where the evolution at the individual level determines the system's ecological organization, and vice versa.

• This study demonstrates an evolutionary feedback between information and organization and, therewith, suggests a potential scenario for the evolution of biological complexity.

## Methods

### Details of the model

As explained before, our model is spatially extended, individual based, Monte Carlo simulation model. One time step of the model consists of a reaction and diffusion step, of which details are described below.

The reaction step was further divided into the production and the decay step. The algorithm of the production step runs as follows. Choose every square of the grid once in a random order. For each chosen square,

(1) if the square contains a free molecule (i.e., a molecule not making a complex; let us denote this by X), then randomly choose another square from the eight squares adjacent to the first square (i.e., Moore neighbors). If the second square also contains a free molecule (denoted by Y), complex formation can happen either between X's 5'-end and Y's 3'-end with a probability $k_{1}=1-\exp(G_{{\text{X}}^{5^{\prime }}\sim^{3^{\prime }}\text{Y}})$ or between Y's 5'-end and X's 3'-end with a probability ${k^{\prime }}_{1}=1-\exp(G_{{\text{Y}}^{5^{\prime }}\sim^{3^{\prime }}\text{X}})$ (*G *is the binding energy between the two dangling-ends as explained in main text). These two possibilities are exclusive. If *k*_1 _+ ${k^{\prime }}_{1}$ > 1, then each probability is normalized by dividing with *k*_1 _+ ${k^{\prime }}_{1}$. If a complex is formed, the two molecules are marked as composing a complex molecule. One complex molecule is represented by two molecules occupying two contiguous squares.

(2) If the square contains a molecule making a complex, then complex dissociation can happen with a probability *k*_2 _= exp(*G*), where *G *is calculated from the two dangling ends forming a bond.

(3) If the square is empty, then chose another square randomly from the Moore neighbors of the first square. If the second square contains a molecule making a complex, then replication can happen with a probability *κ *= 1.

In the decay step, every molecule is removed with a probability *d *= 0.03. If a chosen molecule is forming a complex, then it is removed, but the molecule with which it forms a complex is not removed.

In the diffusion step, every square is chosen once in a random order. For each chosen square, if it contains a molecule, then choose another square from its Moore neighbors. If the second square is empty, the molecule in the first square is moved into the second square with a probability *p*, where *p *= 1 for a free molecule; and *p *= 0.9 for a molecule making a complex (this is to take into account an increased volume of complex). For the diffusion of a complex, the other molecule of the complex that is not in the first square is moved into the first square so that two molecules are always adjacent to each other, and a complex is not chosen twice in one diffusion step.

The model was programed based on CASH library [42]. The source code is available upon request.

### Pre-evolution (preparation for simulations)

From randomly generated RNA sequences, those that can replicate itself were chosen. Homogeneous populations of these replicators were used to initialize a series of simulations. The mutation rate *μ *was initially set to 0.001 or 0.005, but gradually increased to the maximal limit for the survival (i.e., the error-threshold), which was about 0.015. At the end of each simulation, the most abundant genotype was chosen as a pre-evolved replicator. In this way, five different pre-evolved replicators were obtained (see Additional file 3 for the obtained sequences). Then, a homogeneous population of a pre-evolved replicator was used to initialize next simulations, of which results are reported in the paper.

The preparatory evolution was conducted because randomly generated RNA replicators were not able to tolerate high mutation rates used for the main results. Moreover, the fact that each simulation is initialized by a single genotype makes it easier to study the evolutionary dynamics. Therefore, the above preparations enable us to focus on essential results that support our main messages.

### Construction of phylogenies

To construct a phylogeny, two thousand genotypes were selected from the system as follows. At a sufficiently later simulation step (ca. 340000-th steps), the 1000 most abundant phenotypes were chosen each from catalytic and non-catalytic replicators, where a phenotype was defined by the secondary structure in Shapiro's notation [14] and by the sequence of both dangling-ends. For each of those 2000 phenotypes, the genotype with the largest population was chosen to be the representative. Here, one genotype consists of a pair of complementary sequences (for this is the unit of replication); thus, we obtain 2000 pairs of complementary sequences. Complementary sequences must not co-occur in the same phylogeny, so that one has to choose one strand from each pair. This is identical to labeling each strand either as plus or minus (note that this labeling has nothing to do with the functionality of sequences). This labeling was conducted through simulated annealing method that minimizes the Hamming distance among the sequences with the same label. From the so selected 2000 sequences, a phylogeny was constructed by using PHYML [43] with option 0 i 1 0 HKY e e 4 e BIONJ y y. The root of a phylogeny was chosen so as to make the phylogeny more clearly visible. The alignment of sequences was unnecessary because only point mutations were allowed to occur during a simulation. Finally, note that the above simulated annealing method may not necessarily reconstruct the evolutionarily true plus/minus classification if sequence divergence is too large; therefore, the phylogenies constructed here should be considered as a tool to detect the structures in a population of sequences. The validity of this method was checked by a more direct method, in which molecules were labeled either as plus or minus at the beginning of a simulation, and the labels were complementarily copied when replication happens. Phylogenies constructed with this method also detected the same sequence classes reported in this study.

### Construction of sequence logos and typical secondary structures

From the phylogenies shown in Fig. 2, clades representing each sequence class were selected with the aid of TreeDyn [44]. Since each leaf of a phylogeny is a genotype that represents one phenotype, one has to compile, for each selected clade, an ensemble of genotypes in which the population size distribution of genotypes is taken into consideration. From such an ensemble of genotypes, sequence logos were produced by using WebLogo [45], and typical secondary structures were produced in the method described in the caption of Fig. 3.

## Reviewers' comments

### Reviewer's report 1

*Eugene V Koonin, National Center for Biotechnology Information, National Library of Medicine, National Institutes of Health*.

Here Takeuchi and Hogeweg apply Monte Carlo modeling of evolution of "toy" RNA molecules to devise (more or less) general scenario for the evolution of biological complexity. The importance of the problem indisputable. The model is limited in scope because it assumes rather chemically unrealistic requirements for replication and, perhaps, even more importantly, requires "pre-evolution", i.e., the origin of replication, the absolute crucial step in the origin of life, is not addressed. This being said, the results are pretty amazing in that so much complexity and diversity is shown to emerge from very simple rules of replication, interaction, mutation, and decay. It also seems important and intuitively reassuring that compartmentalization emerges as a strict condition of complexification, i.e., the population dies out in a homogeneous medium, just as expected from a biological standpoint. Perhaps, the most striking result is the evolution of parasites and the enhancement of complexification in the presence of parasites. Again, is very biologically plausible, and it is amazing that the effect is seen with such a simple model. With all the limitations of artificial modeling approaches, I think this study tells us something valuable about the earliest stages of life's evolution. I suspect that the model can be readily generalized, i.e., the specific parameters of interaction adopted in this study are not crucial.

#### Authors' response

*We completely agree with the reviewer that the current model does not address the origin of replicators, which is a very important question in RNA world hypothesis*.

*The assumptions made for replication and interactions in our model are indeed chemically unrealistic: First of all, merely having a particular secondary structure does not, in reality, result in the replicase activity. Moreover, base-pair matching between dangling-ends is not the way real RNA-like replicators would interact (for more technical points, please see also our response to Dr. Szathmáry's comments). Thus we agree that the assumed RNA molecules are "toy". However, the current model is, to the authors' knowledge, the first of the kind that explicitly takes account of a biologically relevant genotype-phenotype-interaction mapping of replicators. To achieve this goal, we chose to implement very simple interaction rules*.

*We also think it is important that some kind of spatial isolation is necessary for the maintenance and origin of complexity in replicator systems. In particular, this study dealt with the effect of spatial self-organization, which dynamically segregates different species of replicators, without explicit compartmentalization by lipid or inorganic boundaries*.

*As the reviewer pointed out, an important point of this study is that parasites can actually facilitate the evolution of complexity. It would be very interesting to investigate whether one can extend this idea to other contexts, e.g., to the role of viruses in the evolution of proto-cellular systems*.

### Reviewer's report 2

*Eörs Szathmáry, Collegium Budapest (nominated by Anthony M Poole, Stockholm University)*.

This is an interesting paper that is likely to be cited a lot, and it is likely to trigger similar investigations in the future. I like that the authors stress that information and organization together make complexity, and that the emergent ecosystem creates functionality. I could not agree more.

Whether the details of the concrete model tell us much, beyond its didactic/pioneering value, is another matter. Much depends, for example, on the assumption that during complex formation binding through dangling ends is predefined – we know that this is NOT how, for example, the templates are recognized by the Q*β *(protein!) replicase (since we do not have a replicase ribozyme we have to look at what we have). Very different results may be seen if a certain secondary/tertiary structure were the target to be recognized by the replicase, but the present modelling techniques are not sufficient, so we have to accept the 'toy' approach of the authors.

#### Authors' response

*We recognize that the interaction rules employed in the current model are simplistic. Thus, we must interpret the model and its results with a great care. For this sake, let us speculate what would be similar or different if a model assumes more realistic interaction rules, e.g., by using co-folding of RNA molecules*.

*Firstly, interactions between RNA molecules would still involve base-pair matching as a primary process as in the current rules. However, which parts of two sequences make base-pairs will be determined through complex interactions between the two sequences. This is in contrast to the current rules, where each sequence independently determines which parts can interact with the others (dangling-ends)*.

*Secondly, it still seems reasonable – though possible to conceive otherwise (which is an interesting question in its own sake) – to assume that a replicator has two separate motifs either to recognize or to be recognized by other molecules. If this were the case, then it would still hold that it is more difficult for a catalyst to be recognized by its exact copy than for parasites to be recognized by catalysts because of the problem of intra-molecular recognition in catalysts*.

*Finally, with respect to the evolutionary dynamics, the problem of intra-molecular recognition and the fact that complex formation gives an advantage to parasites – merely to stop replicating the other molecules gives replication advantage *[18] – *can still facilitate the evolution of a parasite and, thereby, that of an escaping catalyst (and so on?). However, such strategies, if they evolve, would be achieved in different, more complex manners than in the current model. Furthermore, more complex ways of interactions might perhaps allow more possible combinations of catalysts and parasites for evolution*.

When talking about complexity, the assumptions of no recombination and of fixed length are presumably rather strong, and one would like to see them reduced in the future. The occupation of sequence/function spaces could be very different as a result of complexification relying not only in point mutations but also on insertions, deletions and recombination (see another remark at the end of these comments).

#### Authors' response

The assumption of fixed sequence length artificially limits the accumulation of information within one quasi-species. We assumed this for simplicity because we wanted to focus on the increase of information via the evolution of diversity. Relaxing this assumption opens up a possibility of complex interplay between these two ways of information accumulation. It is certainly possible that evolutionary dynamics would significantly differ in such a case, and it is indeed interesting future research. (With regard to recombination, please see also our reply to the reviewer's other comment.)

The emerging set of different strategies in relation to the mutation rate is fascinating. When reading main text I thought that the order in which these creatures appear is largely explained by the binding energy, but then in Additional file 2 saw that in the "internally polymorphic" group at high mutation rates the A-catalyst prevailed – so I at the moment do not understand the order clearly. Actually, this functionally polymorphic case is highly interesting, and I suspect that its deeper understanding could result in that of the whole model. Couldn't it be the case that here we are, in contrast to the cases in main text, dealing with 'survival of the flattest' at high mutation rates?

#### Authors' response

*Yes, it is probably the case that because of its "flatness", the A-catalyst of this simulation series wins for greater mutation rates. But why this particular line of replicators develops functional polymorphism without speciation is still not clear to us. While the genotypic diversity of a quasi-species can be explained by its flatness, this functional polymorphism without speciation may be attributed to the topology – rather than the mere flatness – of the local region of the genotype-phenotype mapping in which this line of replicators resides. To facilitate future investigation, we now list the genotype used to initialize this simulation series (and also the other four genotypes) in *Additional file 3.

In the sequentially diverging cases the mutual invasions, and their spatial correlates, are interesting. Yet I am slightly worried about the robustness of these patterns. Based on our re-analysis of Dr. Hogeweg's hypercycle-celullar automaton model, I know that surprisingly little disturbance can collapse mesoscopic patterns. So, what about disturbance of the pattern by a patchy environment, assuming variation in, say, the desorption rate of molecules from the surface across the cells? Actually, in this case I am only slightly worried, but still would feel better if reassured.

#### Authors' response

*This comment led us to examine the effect of spatial heterogeneity in decay rates in the current model. But before describing the results, let us comment on our previous studies of the hypercycle. In our earlier study *[24], *we reported that the hypercycle does not self-organize into spiral-waves if the molecular species in the hypercycle differ too much in their parameters – stated differently, the heterogeneity in individuals disturbs the formation of the spiral-wave patterns. This point was extended in our later study *[7], *which simulated the hypercycle with mutating parameters and showed that the hypercycle is evolutionarily unstable. This study also examined other types of replicator organization than the hypercycle and showed that some of those systems, in particular the ligation system, are stable against parasites despite the continuous mutation of parameters. This difference in the stability is attributed to the difference in the spatial pattern generated by the replicator organization. The spiral wave of the hypercycle is so regularly structured that it is sensitive to the disturbance due to the individual heterogeneity (and to the disturbance due to the spatial heterogeneity as the reviewer pointed out). In contrast, the wave patterns observed in the other replicator organization are more irregular (erratic), and this irregularity probably gives robustness against individual heterogeneity*.

*In the current model, the mutually invading wave pattern is generated by the replicator organization that has arisen through evolution. This means that the pattern has to be robust against individual heterogeneity (otherwise, it would not evolve). Also notable is that the mutually invading wave pattern is erratic and irregular too. Furthermore, this robustness against individual heterogeneity can also bring about the robustness against spatial heterogeneity. To examine this, we studied the case of spatially heterogeneous decay rates in the current model. Following the work referred to by the reviewer *[21], *we randomly placed 100 blocks of size *26 × 26 *with d *= 0.0093 *(d denotes the decay rate) in the grid with d *= 0.03719*. In this way, the decay rate is, on average, the same as the one originally used (d *= 0.03*); moreover, the situation closely matches the case reported in Ref. *[21]* (the same portion of space that has the lower decay rate; the same ratio between the two decay rates). The results of simulations turned out to be qualitatively the same as those presented in this paper*.

I find the terms "species" and "speciation" highly problematic for this model, since there is no recombination. This is not Physica D or E, so I really would recommend dropping this misleading terminology – the term 'sequence classes', used by the authors themselves, is more precise (even if less attractive). Nevertheless, even in the presence of recombination we may find similar diversifying selection on the population, leading to proper speciation, but we cannot know. By the way, the described processes and even some of the plots are similar to what one sees in the field of adaptive dynamics, which also prompts me to point out that the authors are presenting a nice case of frequency-dependent selection. This connection may be spelled out.

#### Authors' response

*Let us first explain why we used the term "species" in this paper. In the current model, a sequence class has an independent line of descent from the other sequence classes after the branching event. Secondly, the sequence classes have distinct ecological functionality. To emphasize these two points, we came to refer to the sequence classes as species*.

*Upon the reviewer's comment, we also examined the effect of recombination. Recombination was implemented in the model by introducing a recombination step, in which every molecule that does not form a complex has the probability (0.01) that it recombines with another non-complex molecule randomly chosen from its Moore neighborhood (the exact algorithm is identical to the other second order reactions). It is assumed that recombination maintains the length of sequences, and it happens independently of the homology between sequences. Simulations were run for the small mutation rates for which four species evolve (ca. 0.004) for the 5 simulation series. In such a set up, recombination was measured to occur on average 0.23 times per individual per average life time of replicators. This is comparable to the probability of mutation per replication, which is 0.18 when μ *= 0.004*. The results showed that speciation still happened despite recombination. In some cases, the four species evolved as we observed in the case without recombination. But there were also differences: (1) It took more time steps for speciation to occur. (2) The sequence of the G-parasite was often more similar to that of the C-catalyst than in the case without recombination. This can be explained by selection to reduce recombination load. (3) The U-parasite often did not evolve. This is probably because the recombination effectively increases the rate of deleterious mutation*.

*In relation to the adaptive dynamics, the current model has an important difference, and we now explain this in Discussion section (please see also our reply to Dr. Adami's comments)*.

*Finally, we note that our examination of recombination is far from a complete investigation, and a more through analysis would be worth while*.

Some other remarks: "authors note that the general concepts discussed above, such as the evolutionary feedback between information and organization, are not entirely new in the field of evolutionary biology." – could we have some citations?

#### Authors' response

*We do not know a literature stating exactly the same concept. But the most closely related literature we know is Ref. *[35], *and we now cite this in Discussion*.

"Spatially extended RNA-like replicator systems can be extremely stable against parasitic replicators, because the system can evolve complex ecological organization that stabilizes itself against strong parasites." What is a 'strong parasite'? Anyway, I think that this conclusion is hardly new.

#### Authors' response

*We realized that the sentence – which originally appeared in the second (currently the third) item of Conclusions – can convey an unintended message. We modified the text*.

"The preparatory evolution was conducted because randomly generated RNA replicators were not able tolerate high mutation rates used for main results" – I think this is a missed opportunity, and it is linked to the idea of starting with smaller molecules and a richer set of component processes (see above).

#### Authors' response

*This is an interesting point as we have already written in this reply. The preparatory evolution was an artificial procedure, in that it merely made use of evolutionary optimization to obtain replicators that can tolerate higher mutation rates. As a future extension of the current model, one can start with shorter RNAs – so that mutation rate per genome is small – and include the possibility of the evolution of longer sequences by allowing more complex chemical/mutational processes*.

### Reviewer's report 3

*Chris Adami and Bjorn Østman, Keck Graduate Institute*.

In this paper the authors study the evolution of RNA-like replicators in a computational system. They observe adaptive radiation and the evolution of co-existence of up to four ecotypes depending on the mutation rate. They conclude that the evolution of complexity in this system relies on an interplay between information acquisition and organization.

This is an interesting study in which ecological complexity is seen to emerge in the absence of a predetermined set of species. Instead, the ecological interactions emerge simply as adaptations to the computational chemistry implemented in the system. The authors suggest that the evolution of parasites (as observed in this study when the mutation rate is low) may also have aided the evolution of complexity in an RNA world, rather than destabilize it.

We have several general remarks and numerous minor points. We hope that the authors can address those in general and in detail, so that our final review will sound much more positive than what we have to say in the following.

Generally speaking, the paper is poorly structured. Results are presented without an apparent ark that orders the observations in support of a testable hypothesis. Several times the reader is promised a result elsewhere in the paper, which then is either not forthcoming or buried. The prose in this paper is very uneven, and drifts from passable via opaque to completely informal comments. This is not the style of a professional research paper. Also, the level of English language proficiency is below an acceptable level, and has to be raised significantly, along with an improvement in orthography.

#### Authors' response

*We improved the text according to the detailed list of linguistic issues painstakingly compiled by the reviewer (the list is not included here), for which we thank the reviewer. With respect to the structure of the paper, we have largely kept the original, because after scrutinizing the text we still think that the original structure best conveys our intended message. We added a paragraph to explain the structure in the presentation of our results (please see the end of Model section)*.

Besides the structural problems of this paper, there are also numerous scientific issues we have to raise. First and foremost among them is the discussion of adaptive radiation. It is beyond doubt that the dynamic observed by the authors is the radiation into several ecoptypes starting from a single (pre-evolved) clone. There is a considerable amount of literature that describes this dynamic both in theory and in simulations as well as in digital life experiments, but the authors seem to be completely ignorant about this vast literature. I am referring to the process of adaptive radiation via negative frequency-dependent selection, as discussed by Doebeli and Dieckmann (see, e, g, Nature 400 (1999) 354). This process has been studied extensively by our group in digital organisms (see Science 305 (2004) 83, and in particular the references to negative frequency-dependent selection in there). Perusing the latter reference will also convince the authors that this is not the first time that the emergence of new species (more properly termed ecotypes) has been observed without a previous explicit determination of those species in a computational system. In fact, it can be argued that this was first seen in Tom Ray's experiments.

#### Authors' response

*We reply to this comment in the reply to the one after the next comment, because the two comments closely hang together*.

In order to test that adaptive radiation has truly created stable ecotypes, you usually need to test that the ecosystem is stable when the mutation rate is reduced to zero. I believe that the authors will see this, but they have not performed the test.

#### Authors' response

*Setting the mutation rate to zero aims to distinguish between the diversity continuously introduced by mutation (as in the genetic diversity within a quasi-species) and the diversity sustained by the ecological functionality of different species. In this study, we established that the diversity evolved in the model is maintained by the ecological difference among species through various methods such as phylogeny, sequence logo and the evolutionary analysis of sequences. Furthermore, setting the mutation rate to zero in the current model causes the side effects that directly affect the interactions among species, namely, effective dilution and greater catalyst diversity (please see also "Effect of the mutation rate on the evolution"). These side effects make this test ambiguous. Because of these two reasons, we did not use this test in the current study (but please see below). Finally, we would like to mention that such side effects – narrowing the "width" of quasi-species distribution by setting mutation rate to zero affects the population dynamics at ecological time scale – can happen in wider classes of ecological systems (e.g., see van der Laan and Hogeweg (1994) Proc R Soc Lond B 259:35)*.

*Upon the reviewer's comment, we nevertheless examined the case of zero mutation rate. Simulations were conducted for μ *= 0 *by continuing the simulations in which four species evolved. Somewhat to our surprise, one case (out of the 5 different simulation series) showed the coexistence of four *genotypes. *The ecological function of the four genotypes is interpretable as the dominant catalyst, the parasite, the escaping catalyst and the parasite to the escaping catalyst, as in the system of the C- and the A-catalyst and the G- and the U-parasite. The four genotypes are, however, much less characterized by a single nucleotide type. Apart from this case, three genotypes remained in one case, and two genotypes remained in three cases. In these latter four cases, the ecological functionality of the surviving genotypes can be characterized in a similar manner too. But their sequences are often not dominated by a single nucleotide type either, and their dangling-ends are not similar to each other among the different simulation series. From these results, an interesting observation can be made: The mutation causes more extreme base composition in the evolving genotypes, making the genotypes of different species distinct. Interestingly, this actually makes the evolving phenotypes very similar among the different runs of evolutionary dynamics*.

We also have to strenuously object to the assertion, expressed on p. 7 (*Authors' note: this refers to the first paragraph of the section titled "Ecological organization of the replicator system"*), that the C-catalyst and the A-catalyst are exploiting the same resource (identified as "empty space"). This comment underscores the extreme deficiencies in understanding the process of adaptive radiation. Due to the competitive exclusion principle (also known as Gause's principle), this is strictly forbidden. If two ecotypes exploit the same resource, then they compete directly and only one can survive. Specifically, turning off mutation will result in the emergence of a single clone. It is quite clear that the limiting resource for the C-and the A-catalyst is the presence of C- and A-catalysts themselves. The represent different (limited) resources, and thus they can coexist. Note that the waves that the authors see are just those predicted from "invasion when rare". Note also that the destruction of the ecosystem when the population is well-mixed is also observed in the bacterial experiments of Rainey and Travisano, Nature 394 (1998) 69. (However, this is not necessary, as seen in Chow et al., Science 305 (2004).)

#### Authors' response

*First of all, we would like to explain that the coexistence mechanism suggested by the reviewer is incorrect. While the catalyst may be considered as the resource of itself in the sense that it is the "catalytic (and template) resource", this type of resource crucially differs from the resource in the usual sense of the word. Usually, the resource is depleted as a population grows as is the case in the reviewer's study *[34]. *This means that there is a negative feedback between the size of the population and the abundance of the resource. But the resource in the sense of catalysts and templates is not depleted, but actually increases, as the catalyst population grows. Thus, there is a positive feedback between the population size and the abundance of the resource. Therefore, using a different catalytic resource cannot by itself enables the coexistence of different species. Instead, it is more helpful to see that all replicators compete for one and the same resource (empty space). From this point of view, we explained that the coexistence of the two catalytic species were mediated by the preferential parasitism and spatial pattern formation*.

*Related to the above argument is the crucial difference between the diversification happening in the current model and that in the models cited by the reviewer. In the adaptive dynamics model of Doebeli and Dieckmann *[33]* and the Avida model of the reviewer *[34], *the diversification happens as the radiation of a population into the space of predefined ecological functionality, where having sufficiently different functionality – i.e., consuming different resources – enables a species to coexist with the others. In contrast, the diversification in the current model happens through a chain reaction of niche generation and speciation. The ecological functionality in the current model, such as a parasite and an escaping catalyst, is not predefined, but is generated by the evolved ecological organization. To explicitly explain this crucial point, we have modified main text (especially in Discussion)*.

*Secondly, the reviewer implies that the effect of space in the current model is parallel to that in Rainey and Travisano's experiment. Although spatial heterogeneity was crucial for the diversification in both studies, there is an important difference between the two. While in Rainey and Travisano's experiment the environment is externally set to be spatially heterogeneous (the title of their paper), in the current model there is no preexisting environmental spatial heterogeneity. Instead, a population self-organizes into spatial patterns, and it is this spontaneous pattern formation that is crucial to the diversification (and maintenance) of the current system*.

*Thirdly, we should add a few words on the competitive exclusion principle. Firstly, a strict contradiction to the principle exists as shown by Koch (J Theor Biol (1974) 44:387–397). Moreover, the principle ceases to hold if one takes predation into account as shown by Cramer and May (J Theor Biol (1972) 34:289–293), which is known as the predator-mediated coexistence (Caswell (1978) Am Nat 112:127–154). The current model gives another such example of coexistence mediated by the processes at other ecological levels*.

*Finally, upon the reviewer's comment on Tom Ray's Tierra, we added a paragraph to explicitly acknowledge his study in relation to the current study*.

The authors make an intriguing observation that seems to contradict the paradigm of "survival of the flattest" (or, evolution of mutational robustness). Again, no references to a large and mature literature in this field, starting with van Nimwegen, and continuing with Wilke et al (Nature 2001) and other references by Wilke, are made.

Besides the fact that the observation is made on p. 7 ("why this is the case will become clear later"), and there is only a single cryptic sentence at the bottom of p. 10 that we could find that refers to this, the violation of mutational robustness is very unclear. The sentence "Too high mutation rates make the G-parasite too weak to enable the A-catalyst to out-compete the C-catalyst" does not shed any light on this mechanism at all. In fact, the entire section "Dependence of speciation on mutation rate" is very weak, with almost no mechanistic explanation. I think that the dynamics that lead to an apparent violation (and we insist "apparent", because of course this principle cannot be violated) of the mutational robustness paradigm are potentially very interesting, because it points to the mutualistic dependence on another genotype whose robustness needs to be taken into account.

#### Authors' response

*Let us begin with a general remark. Firstly, the evolution of mutational robustness does not happen if the product of the population size and the mutation rate is too small as shown in *Fig. 3* of van Nimwegen *et al. *(PNAS (1999) 96:9716). Secondly, the survival of the flattest does not happen for a given mutation rate if the replication rate of the flattest is too small compared with that of the fittest as shown in *Fig. 3(a)* of *[17]. *This latter point explains why the A-catalyst does not evolve instead of the C-catalyst for higher mutation rates. We modified the text of "Effect of the mutation rate on the evolution" to explain this more clearly. We now also cite the studies by Eigen *et al. [15]* (their Fig. 17 is of particular relevance) and by Wilke *[17]* in main text*.

A few observations on the calculation of informational complexity. The numbers determined in the caption to Figure 3 are never used in the text, even though a number of interesting observations can be made using those numbers and the survival of the flattest effect. However, the author would have to quantify the fitness of the catalysts and parasites, which is in principle possible (but was not carried out). The observation that informational complexity cannot be determined for an ecosystem is correct, and constitutes a major problem. There are ways in which the measure can be extended to cover different niches but overlapping niches can still be a problem. This study clearly shows the need for a mathematically sound extension of this measure, which is a problem we have been working on for a while now.

#### Authors' response

We are glad to share the view on the problem of physical complexity. What exactly are the possible interesting observations?

The observation concerning "functional polymorphism without speciation" is interesting, but it is not clear why it is buried in an additional file. Again, this reflects poorly on the structure of this paper. That the observed radiation is actually a polymorphism can be shown by setting the mutation rate to zero. The authors should do this.

#### Authors' response

*First of all, we would like to note that this polymorphism happens within one quasi-species without speciation. Thus, this polymorphism is different from the diversification associated with speciation as observed in the other simulation series*.

*Nevertheless, we also examined the case of μ *= 0 *in the system with polymorphism without speciation. The results showed that four genotypes survived, where two genotypes are catalytic, while the other two are non-catalytic (parasitic). The mechanism of their coexistence seems to be similar to that of the four species evolving in the simulations with small mutation rates, in that one parasitic genotype has slight preference to one catalyst both in its affinity to the catalyst (G) and in its spatial distribution; so does the other parasite to the other catalyst. The system, however, does not clearly exhibit mutually invading wave patterns. To facilitate future investigation, we now list the genotype used to initialize this simulation series and also the other four genotypes in *Additional file 3.

Finally, we are missing an analysis of the evolutionary dynamics along the line of descent, as is now standard in computational evolution.

#### Authors' response

*Because of computational overload, we used the phylogenetic method. To infer the evolutionary relationship, the ancestor tracing is of course the best, and it can give much more information. But, since we were able to make a sufficient analysis of evolutionary dynamics through the other methods for our sake, we did not perform the ancestor tracing*.

A few more points:

1. In a system with, say, 6 nucleotides, could 6 phenotypes evolve? If the ecosystem is limited by the number of nucleotides, then it is not clear whether the observation of an emergent ecosystem has anything to do with the complexity of an RNA world, in particular because we assume that the mutation rate is high, and no ecosystem evolves at high rates.

#### Authors' response

*We do not know if 6 phenotypes evolve. We, however, would like to note that it was unexpected that the evolved sequence strategies specialized on specific nucleotide types. If the mutation rate is much smaller than those used in this paper, the strategies of catalysts and parasites are not anymore simply describable as C or G, because the sequence patterns temporally keep on changing (however, the results in this parameter region are not reported in detail)*.

*Furthermore, the specialization of species on specific nucleotide types is due to the simple interaction rules employed in the current model, and thus we do not claim that exactly the same sequence strategies would evolve in the RNA world. If more complex interaction rules are employed, we expect that the results would differ (please see also our reply to Dr. Szathmáry's comment)*.

*The current study showed that high mutation rates prohibit the evolution of complex ecological organization. This could be a potential problem for the evolution of complexity in prebiotic evolution in addition to the error-threshold*.

2. Figure 2(a) has many dangling ends with a majority of both C's and G's, suggesting both C-catalysts and C-parasites. Yet it is described as only consisting of C-catalysts. The same goes for some of the cluster in the (b), (c) and (d), also with A-catalysts and U-parasites mingling. To what degree do C-catalysts "tend" to appear cyan, etc?

#### Authors' response

*The non-catalytic genotypes in *Fig. 2(a)* do not constitute a separate parasitic lineage, but are members of the quasi-species of the C-catalyst. To clarify this point we have added explanation in several places throughout main text. For the color coding, we added insets and more explanation in *Fig. 2.

3. It would also be nice with a short explanation of what "secondary structure in Shapiro's notation" means. Also, why not plot the secondary structure of the sequences in Fig. 3? We tried to first construct them from the parenthesis notation, and after this did not work out we figured we'd plot them using the RNAplot routine of the Vienna package, but it did not install properly on the Mac. We don't think this is too much to ask.

#### Authors' response

*An example of the catalytic secondary structure is shown in *Fig. 1. *We now mention this fact in main text*.

4. What is the meaning of "temporarily unstable"? (Additional file 2) Does it re-enter a period of stability after having been unstable for a while?

#### Authors' response

*One of the parasite species dies out once in a while, but the secondary parasite recurrently evolves during the same simulation*.

5. It would be nice to see an animation of the wave-fronts as in Figure 4. This would be a good use of "additional files".

#### Authors' response

*We added a movie file *(Additional file 1).

6. Figure 6(e). By what number does the number of species fluctuate for intermediate mutation rates?

#### Authors' response

*The polymorphic catalyst species is counted as one species. There can be one or two parasite species. Thus, the number fluctuates between 2 and 3*.

In general, do not use the word "duality". There is really no meaning in this word here. Use "interplay between information and robustness" instead. The first sentence of the results section really is completely empty as it stands (although we know what the authors mean by it).

#### Authors' response

We deleted the word "duality of information and organization." However, instead of the suggested expression, we now use "interdependence of information and organization."

## Competing interests

The author(s) declare that they have no competing interests.

## Authors' contributions

NT and PH conceived of the study, designed the model, interpreted the results and revised the manuscript. NT programmed the model, analyzed the data and wrote the first draft of the manuscript. Both authors read and approved the final manuscript.

## Supplementary Material

Additional file 1 **Movie of a simulation**. This file is a MPEG movie of a simulation for *μ *= 0.004. The color coding is the same as in Fig. 4. Click here for file

Additional file 2 **Functional polymorphism without speciation**. This file explains the results of the simulation series that showed a functional polymorphism without speciation (see also Fig. 6). It also discusses its implication for the measurement of physical complexity (see also Discussion). Click here for file

Additional file 3 **Five genotypes used to initialize simulations**. This file lists the five catalytic genotypes obtained from pre-evolution. They are used to initialize simulations. The first one is for the simulations presented in main text. The last one is for the simulations presented in Additional file 2 Click here for file

## References

[B1] Schuster P, Fontana W, Stadler PF, Hofacker IL (1994). From sequences to shapes and back: a case study in RNA secondary structures. Proc R Soc Lond B.

[B2] Stadler BMR, Stadler PF (2004). Molecular replicator dynamics. Adv Complex Syst.

[B3] Szathmáry E (2006). The origin of replicators and reproducers. Philos Trans R Soc Lond B.

[B4] Gesteland RF, Cech TR, Atkins JF, (Eds) (2006). The RNA world.

[B5] Eigen M, Schuster P (1979). The hypercycle – a principle of natural self-organization.

[B6] Eigen M (1971). Selforganization of matter and the evolution of biological macromolecules. Naturwissenschaften.

[B7] Hogeweg P, Takeuchi N (2003). Multilevel selection in models of prebiotic evolution: compartments and spatial self-organization. Orig Life Evol Biosph.

[B8] Happel R, Stadler PF (1998). The evolution of diversity in replicator networks. J Theor Biol.

[B9] Manrubia SC, Poyatos JF (2003). Motif selection in a model of evolving replicators: The role of surfaces and limited transport in network topology. Europhys Lett.

[B10] Forst CV (2000). Molecular evolution of catalysis. J Theor Biol.

[B11] Altmeyer S, Füchslin RM, McCaskill JS (2004). Folding stabilizes the evolution of catalysts. Artif Life.

[B12] Attolini CSO, Stadler PF (2006). Evolving towards the hypercycle: A spatial model of molecular evolution. Physica D.

[B13] Hofacker IL, Fontana W, Stadler PF, Bonhoeffer LS, Tacker M, Schuster P (1994). Fast folding and comparison of RNA secondary structures. Monatsh Chem.

[B14] Shapiro BA (1988). An algorithm for comparing multiple RNA secondary structures. Comput Appl Biosci.

[B15] Eigen M, McCaskill J, Schuster P (1989). The molecular quasi-species. Adv Chem Phys.

[B16] Schneider TD, Stephens RM (1990). Sequence logos: a new way to display consensus sequences. Nucleic Acids Res.

[B17] Wlke CO (2001). Selection for fitness versus selection for robustness in RNA secondary structure folding. Evolution Int J Org Evolution.

[B18] Takeuchi N, Hogeweg P (2007). The role of complex formation and deleterious mutations for the stability of RNA-like replicator systems. J Mol Evol.

[B19] Kaneko K, Ikegami T (1992). Homeochaos: dynamic stability of a symbiotic network with population dynamics and evolving mutation rates. Physica D.

[B20] Ray TS (1994). Evolution, complexity, entropy and artificial reality. Physica D.

[B21] Scheuring I, Czárán T, Szabó P, Káarolyi G, Toroczkai Z (2003). Spatial models of prebiotic evolution: soup before pizza?. Orig Life Evol Biosph.

[B22] Szathmáry E, Demeter L (1987). Group selection of early replicators and the origin of life. J Theor Biol.

[B23] Boerlijst MC, Hogeweg P (1991). Spiral wave structure in pre-biotic evolution: Hypercycles stable against parasites. Physica D.

[B24] Boerlijst M, Hogeweg P, Langton CG, Taylor C, Farmer JD, Rasmussen S (1991). Self-structuring and selection: Spiral waves as a substrate for prebiotic evolution. Artificial Life II.

[B25] Maynard Smith J (1979). Hypercycles and the origin of life. Nature.

[B26] Bresch C, Niesert U, Harnasch D (1980). Hypercycles, parasites and packages. J Theor Biol.

[B27] Hanczyc MM, Dorit RL (1998). Experimental evolution of complexity: in vitro emergence of intermolecular ribozyme interactions. RNA.

[B28] Manrubia SC, Briones C (2007). Modular evolution and increase of functional complexity in replicating RNA molecules. RNA.

[B29] Niesert U, Harnasch D, Bresch C (1981). Origin of life between Scylla and Charybdis. J Mol Evol.

[B30] Koch AL (1984). Evolution vs the number of gene copies per primitive cell. J Mol Evol.

[B31] Koonin EV, Martin W (2005). On the origin of genomes and cells within inorganic compartments. Trends Genet.

[B32] van der Laan JD, Hogeweg P (1994). Predator-prey coevolution: Interactions across different timescales. Proc R Soc Lond B.

[B33] Dieckmann U, Doebeli M (1999). On the origin of species by sympatric speciation. Nature.

[B34] Chow SS, Wilke CO, Ofria C, Lenski RE, Adami C (2004). Adaptive radiation from resource competition in digital organisms. Science.

[B35] Ray TS, Langton CG, Taylor C, Farmer JD, Rasmussen S (1991). An approach to the synthesis of life. Artificial Life II.

[B36] Maynard Smith J, Szathmáry E (1997). Major Transitions in Evolution.

[B37] Hazen RM, Griffn PL, Carothers JM, Szostak JW (2007). Functional information and the emergence of biocomplexity. Proc Natl Acad Sci USA.

[B38] Lynch M (2007). The frailty of adaptive hypotheses for the origins of organismal complexity. Proc Natl Acad Sci USA.

[B39] Moran NA (2007). Symbiosis as an adaptive process and source of phenotypic complexity. Proc Natl Acad Sci USA.

[B40] Adami C, Ofria C, Collier TC (2000). Evolution of biological complexity. Proc Natl Acad Sci USA.

[B41] Szostak JW (2003). Functional information: Molecular messages. Nature.

[B42] de Boer RJ, Staritsky AD CASH. http://theory.bio.uu.nl/rdb/software.html.

[B43] Guindon S, Gascuel O (2003). A simple, fast, and accurate algorithm to estimate large phylogenies by maximum likelihood. Syst Biol.

[B44] Chevenet F, Brun C, Banuls AL, Jacq B, Christen R (2006). TreeDyn: towards dynamic graphics and annotations for analyses of trees. BMC Bioinformatics.

[B45] Crooks GE, Hon G, Chandonia JM, Brenner SE (2004). WebLogo: a sequence logo generator. Genome Res.

