# Supplementary material for: Evolution of complexity in RNA-like replicator systems
Source: Biol Direct. 2008 Mar 27;3:11. doi: 10.1186/1745-6150-3-11 (PMC2390529; doi:10.1186/1745-6150-3-11)
Supplement: Additional file 2 — Functional polymorphism without speciation. This file explains the results of the simulation series that showed a functional polymorphism without speciation (see also Fig. 6). It also discusses its implication for the measurement of physical complexity (see also Discussion). [file 1745-6150-3-11-S2.pdf]

# Additional File 1 for "Evolution of complexity in RNA-like replicator systems"

N. Takeuchi
P. Hogeweg

## 1 Functional polymorphism without speciation

As we mentioned in main text, one of the simulation series deviates from the other four series in some aspects. In this document we explain the results of this atypical simulation series and also discuss its implication for the measurement of physical complexity [1], which is discussed in main text (see Discussion).

The system in this simulation series differs from the others in two aspects, which might be related to each other: the phenotype of the catalytic species that evolves for high mutation rates and the presence/absence pattern of sequence classes as a function of the mutation rate. For very high mutation rates ($\mu = 0.013, 0.014$), the system evolved only one catalytic species, which is the same as in the other series. However, this catalytic species turned out to be the A-catalyst, which contrasts with the other cases where it was the C-catalyst (the evolution of the A-catalyst can be explained by survival of the flattest effect; see also Authors' response to Reviewer's report 2). For lower mutation rates ($\mu \leq 0.012$), a parasitic species evolved, which made the catalyst to evolve functional polymorphism without speciation as we will describe in more detail in the next paragraph. In this region of the parameter, the number of evolved sequence classes fluctuate; namely, the system contains one catalyst class and one or two parasite classes. This pattern of sequence class composition differs from what we observed in the other series. If the mutation rate was even lower ($\mu = 0.003, 0.002$), the catalyst speciated to the typical C- and A-catalyst as in the other series, and the parasite population also speciated into the typical G- and U-parasite species. Hence the four-species ecosystem evolved for low mutation rates as in the other simulation series. Therefore, the ecological organization with four species is a robust evolutionary attractor of the current model.

Fig. 7(a) shows a phylogeny constructed from a population of sequences in the system for $\mu = 0.01$ . As seen from the phylogeny, there are one catalytic class and two parasitic classes in the system. Interestingly, the 5'-dangling-end of the catalytic class contains C's and A's in a nearly equal frequency as seen from the sequence logo in Fig. 7(b) (let us call this class AC-catalyst). This, however, does not mean that each individual of the AC-catalyst has such a sequence composition. In fact, the close inspection of the phylogeny reveals that some genotypes of the AC-catalyst have more C's in their 5'-dangling-end, while the others have more A's (i.e., some leaves of the phylogeny are cyan, while the others are magenta). Therefore, the AC-catalyst holds both C- and A-catalyst like sequence strategies within one quasi-species (without speciation). In addition, we note that the two parasitic classes differ greatly in sequence and structure; however, the target of parasitism is not very well discriminable. Moreover, the system does not exhibit wave-like patterns as shown in Fig. 7(c).

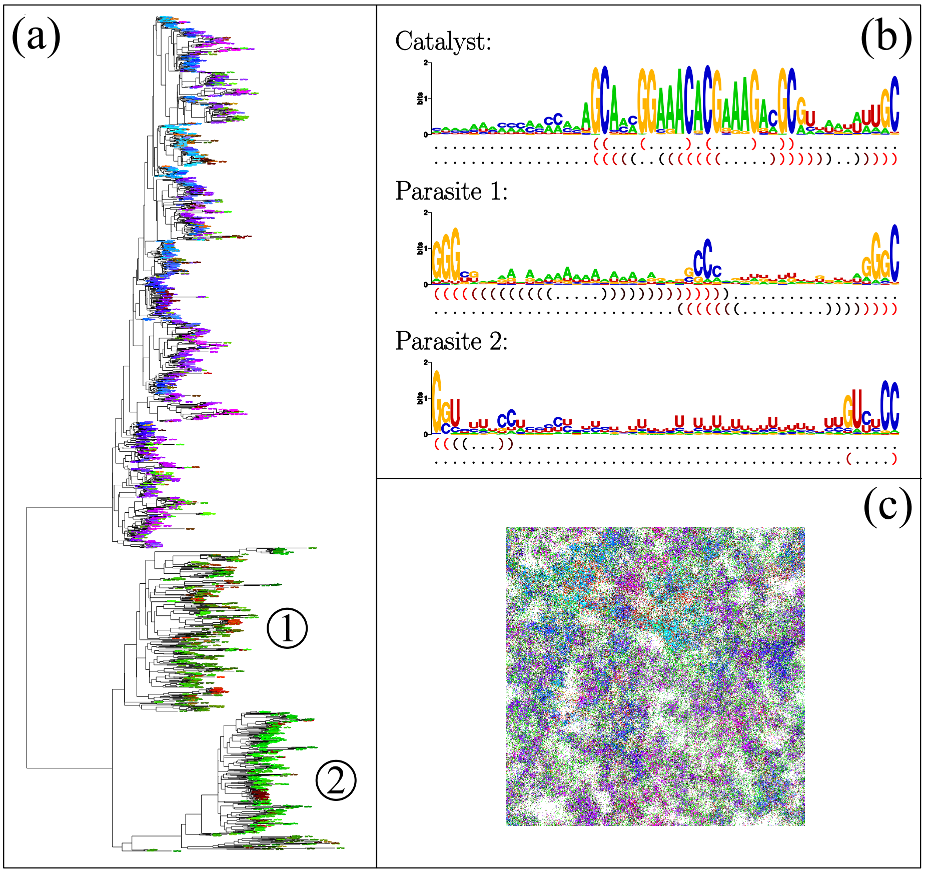


Figure 7: This figure shows the results from one of the 5 simulation series that exhibited the functional polymorphism within quasi-species in the catalysts. The results shown here were obtained from a simulation with $\mu = 0.01$. **(a)** Phylogeny. The method of construction and the color coding of leaves are the same as in Fig. 2. The numbers next to clades correspond to those of the sequence logos of the parasites. **(b)** Sequence logos, constructed in the same way as in Fig.3. **(c)** A snapshot of simulation. The color coding is the same as in Fig. 4.

The fact that two parasitic classes appear in the system strongly indicates that the above sequence polymorphism in the catalyst has a significant impact on the evolutionary dynamics of parasites. Although the coexistence of the two parasitic classes turns out to be not stable in long run (one of the two parasitic classes goes extinct), the evolution of a secondary parasite happens recurrently during one simulation (data not shown).

Finally, with respect to the discussion of physical complexity in main text, the above results exemplify a case in which it is difficult to make one-to-one correspondence between phylogenetic and functional classes. In contrast to the previous case in Fig. 3, the classification of sequences through the phylogeny cannot take account of the functional polymorphism of the catalysts, because the polymorphism appears without speciation in the current case.

# References

[1] Adami, C, Ofria, C, Collier, TC: **Evolution of biological complexity**. *Proc Natl Acad Sci USA* 2000, **97**:4463-4468.
